# Cathepsin G is associated with cerebral vascular injury in myeloid leukemia: a pathologic insight into intracranial hemorrhage

**DOI:** 10.1016/j.rpth.2026.103433

**Published:** 2026-03-26

**Authors:** Toshihiro Gi, Kaiyou Kai, Kotaro Shide, Eriko Nakamura, Nobuyuki Oguri, Murasaki Aman, Kazunari Maekawa, Sayaka Moriguchi-Goto, Michikazu Nakai, Kazuya Shimoda, Yohei Hisada, Atsushi Yamashita

**Affiliations:** 1 Department of Pathology, Faculty of Medicine, University of Miyazaki, Miyazaki, Japan; 2 Division of Hematology, Diabetes, and Endocrinology, Department of Internal Medicine, Faculty of Medicine, University of Miyazaki, Miyazaki, Japan; 3 Department of Diagnostic Pathology, University of Miyazaki Hospital, Faculty of Medicine, University of Miyazaki, Miyazaki, Japan; 4 Department of Statistics and Data Management, Faculty of Medicine, University of Miyazaki, Miyazaki, Japan; 5 UNC Blood Research Center, Division of Hematology, Department of Medicine, University of North Carolina at Chapel Hill, Chapel Hill, North Carolina, USA

**Keywords:** autopsy, brain, cathepsin G, hematologic neoplasms, intracranial hemorrhage

## Abstract

**Background:**

Intracranial hemorrhage (ICH) is a fatal complication of leukemia; however, mechanisms underlying its development, particularly central nervous system (CNS) involvement and vascular injury, remain unclear.

**Objectives:**

We aimed to investigate the histopathologic features of cerebral vessels in leukemia and the expression of hemostasis-related factors in leukemia cells.

**Methods:**

We conducted an autopsy-based study including 37 leukemia cases and 20 matched controls. Histopathologic analysis of CNS tissues was performed to evaluate ICH, leukemia cell localization, and vascular injury. Immunohistochemistry was performed to assess expression of vascular endothelial growth factor (VEGF), cathepsin G, tissue-type plasminogen activator, urokinase-type plasminogen activator, urokinase-type plasminogen activator receptor, and tissue factor in leukemia cells. Vascular integrity was evaluated using stains for smooth muscle actin, collagen, fibrin, and von Willebrand factor.

**Results:**

ICH was identified in 68% of leukemia cases and was associated with fatal brain herniation in 40%. CNS involvement was observed in 54% of cases, often without a clinical diagnosis. The leukemia cell infiltration of meninges and vascular walls was frequently associated with changes in smooth muscle cells and adventitial collagen. CNS vascular injury was frequently associated with ICH in the presence of leukemia cell infiltration. VEGF and urokinase-type plasminogen activator were highly expressed in leukemia cells. VEGF was associated with meningeal invasion, while cathepsin G was predominantly expressed in myeloid leukemia and linked to vascular damage.

**Conclusion:**

VEGF and cathepsin G may serve as markers of meningeal invasion and cerebral vascular damage in leukemia, respectively.

## Essentials

• Intracranial hemorrhage is a fatal complication of leukemia.
• This autopsy-based study assessed cerebral vascular injury and factors expressed by leukemia cells.
• Cerebral vascular injury was accompanied by leukemic cell invasion within vascular walls.
• Cathepsin G and VEGF were associated with vascular injury and meningeal invasion, respectively.

## Graphical abstract

**Figure:**
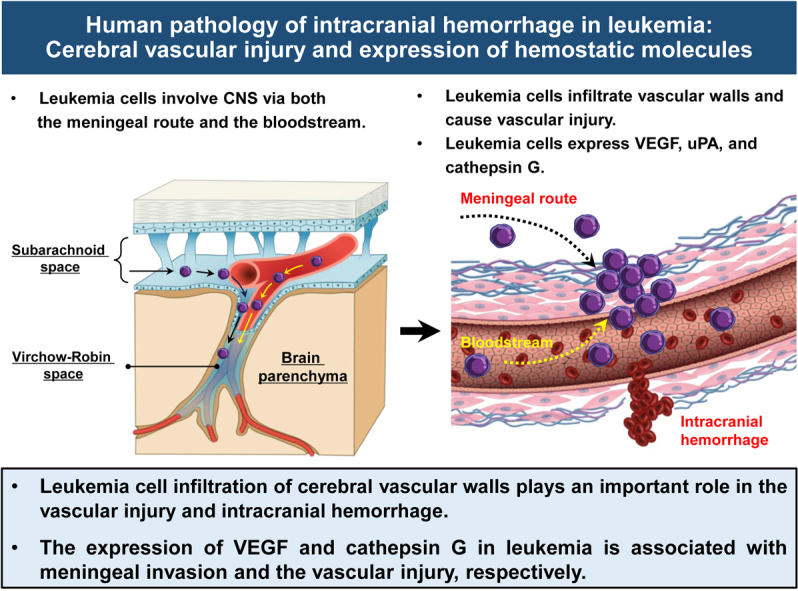

## 1 Introduction

Intracranial hemorrhage (ICH) is a life-threatening complication in patients with hematological malignancies, particularly leukemia. In acute leukemia, the early mortality rate of patients who develop ICH was previously reported to be as high as 64% [1], highlighting the importance of elucidating mechanisms underlying this complication. Clinical studies investigated ICH in acute leukemia and identified a number of risk factors, including age, sex, leukemia subtype, white blood cell (WBC) count, platelet count, and prothrombin time (PT) [[2], [3], [4], [5], [6]].

The majority of mechanistic studies have been conducted using leukemia murine models and peripheral blood from leukemia patients. These studies indicated the contribution of cytokines and hemostasis-related factors expressed by leukemia cells to coagulation abnormalities, activation of fibrinolytic pathways, and vascular endothelial damage [[6], [7], [8], [9], [10], [11]]. These molecular abnormalities, together with thrombocytopenia caused by bone marrow suppression and an increased risk of infection, are considered to contribute to a complex bleeding diathesis. Therefore, ICH may predominantly be attributed to systemic bleeding tendency rather than a regional change in central nervous system (CNS).

The local histopathology of hemorrhagic lesions in humans has not been examined in detail. Autopsy-based studies described ICH in leukemia patients and noted CNS involvement [[12], [13], [14], [15], [16], [17]]. The pathologic findings of leukemia with ICH were identified as leukostasis, defined as dilated cerebral vessels densely packed with leukemia cells, and leukemic nodules, which are dense clusters of leukemia cells within the brain parenchyma [12]. However, a detailed pathologic evaluation of the affected vasculature and expression of hemostatic factors in leukemia cells has not yet been performed. Therefore, the relationship between vascular infiltration and hemorrhage remains unclear.

Hemostasis-related factors derived from leukemia cells may contribute to hemorrhagic complications. Vascular endothelial growth factor (VEGF), which increases vascular permeability, was shown to be expressed in leukemic blasts in both peripheral blood and bone marrow [18]. Brain tumor–derived VEGF can compromise vascular function and promote hemorrhage in a murine model [19]. Furthermore, cathepsin G, a serine protease highly expressed in leukemia, induced endothelial injury in *in vitro* cell models [20,21]. Elevated levels of fibrinolysis-related factors have also been detected in the peripheral blood of patients with acute leukemia and have been associated with bleeding tendency [22]. Based on these findings, we hypothesized that expression of these factors in leukemia cells may contribute to the development of ICH.

Therefore, the present study investigated histopathologic changes in cerebral vessels in human autopsy specimens to clarify expression of hemostasis-related factors in leukemia cells, the direct involvement of leukemia cells in ICH, and their potential impact on vascular integrity.

## 2 Methods

### 2.1 Selection of leukemia cases

This retrospective study was approved by the Ethics Committee of the University of Miyazaki (protocol number: O-1417). The study design is summarized in Supplementary Figure 1. We selected leukemia cases (*n* = 53) from consecutive autopsy cases at the University of Miyazaki Hospital between 1977 and 2023 (*N* = 2673). Sixteen cases without a CNS examination were excluded. We also selected autopsy cases without CNS diseases for the control group (*n* = 20), matched by age and sex with the leukemia group. Cases in the control group had no history of hematological malignancies or CNS diseases, such as stroke, cancer metastasis to the CNS, neurodegenerative diseases, or CNS infections. We analyzed the clinicopathologic backgrounds, histologic types of leukemia, and presence of ICH based on autopsy records. Laboratory data were obtained from blood samples collected closest to the day of death. Systemic bleeding tendency was defined as the presence of macroscopic and microscopic hemorrhages in multiple organs, based on autopsy findings. We then performed a histopathologic analysis of available hematoxylin and eosin (HE)-stained brain specimens. We also conducted immunohistochemistry and immunofluorescence using paraffin-embedded tissues of the brain and systemic organs.

### 2.2 Histologic analysis with HE-stained sections

**Figure 1:**
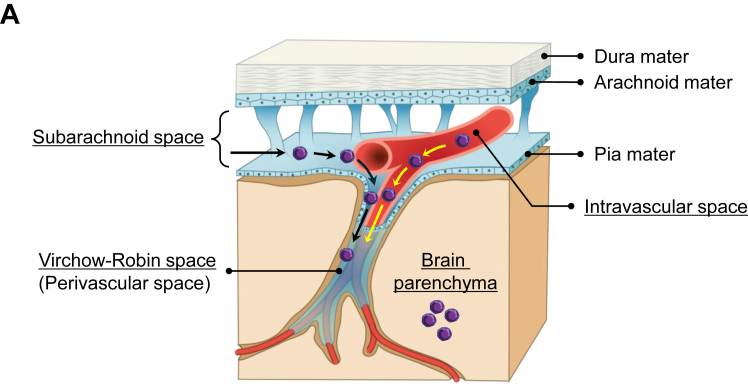

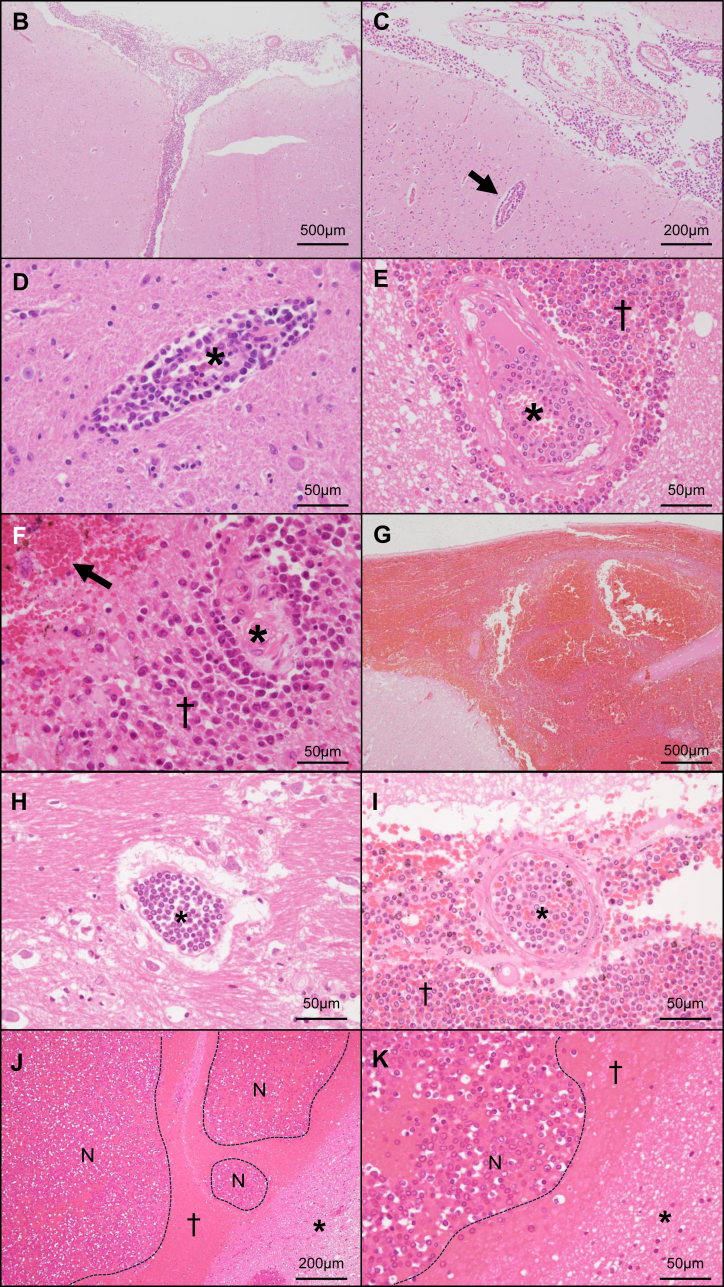
Patterns of central nervous system (CNS) involvement in leukemia and intracranial hemorrhage. (A) Schematic representation of the meningeal structure and brain vessel distribution. Leukemia cells infiltrate the CNS via cerebrospinal fluid or blood flow. Leukemia cells disseminated through cerebrospinal fluid flow extend into the subarachnoid space and Virchow–Robin space (VRS). In this study, the localization of leukemia cells was classified as follows: (1) the subarachnoid space extending into the VRS, (2) the intravascular space, and (3) the brain parenchyma. (B–D) Pathologic patterns of CNS involvement in leukemia. (B) Leukemia cell infiltration in the subarachnoid space in a case of acute lymphoblastic leukemia, consistent with meningeal invasion. (C) Leukemia cell infiltration along the subarachnoid space and VRS (arrow) in a case of acute myeloid leukemia (AML). (D) Leukemia cell infiltration in the VRS in a case of AML. Leukemia cells are localized in the VRS without brain parenchymal involvement. The asterisks indicate the vascular lumens (D and E). (E–G) Pathologic patterns of intracranial hemorrhage. (E) Subarachnoid hemorrhage in a case of chronic myeloid leukemia in the blast phase (CML-BP) with fatal brain herniation. Hemorrhage extends into the VRS (dagger), accompanied by dense leukemia cell clusters. Leukemia cells are also present in the intravascular space (asterisk). (F) Brain parenchymal hemorrhage in a case of AML. A vessel is indicated by an asterisk. Presence of leukemia cells in the VRS and parenchyma (dagger), accompanied by hemorrhage (arrow). (G) Subarachnoid hemorrhage in a case of AML with fatal brain herniation. (H, I) Pathologic leukostasis in brain vessels in cases of AML (H) and CML-BP (I). Intravascular spaces are filled with leukemia cells, with few or no erythrocytes visible (H and I, asterisks). (I) A vessel with leukostasis is observed within a subarachnoid hemorrhagic site infiltrated by leukemia cells (dagger). (J, K) Leukemic nodules in intracranial hemorrhagic sites in a case of AML. In the low-magnification field (J), multinodular, hypercellular lesions (*N*, within dashed lines) are observed within a hemorrhagic area (dagger). In the high-magnification field (K), these nodular lesions (*N*) consist of densely packed leukemia cells. Asterisks indicate glial tissue (J and K).

All specimens were fixed in 20% to 30% formaldehyde, embedded in paraffin, sectioned at a thickness of 2.5 μm, and stained with HE. The presence and distribution of leukemia cells in brain tissue were analyzed. The anatomical sites of leukemia cell localization were classified as follows: the subarachnoid space extending to the Virchow–Robin space, the intravascular space, and the brain parenchyma (Figure 1A). CNS involvement was defined as the presence of leukemia cells in any of these sites. Infiltration within the subarachnoid space extending to the Virchow–Robin space was regarded as meningeal invasion by leukemia. We also investigated whether pathologic leukostasis and leukemic nodules were present, as previously described [12]. Briefly, pathologic leukostasis was defined as dilated cerebral vessels densely packed with leukemia cells and leukemic nodules as dense clusters of leukemia cells within the brain parenchyma [12].

The sites of ICH were classified as follows: the subarachnoid space extending to the Virchow–Robin space, the brain parenchyma, the intraventricular space, and the subdural region. The causes of ICH were examined using autopsy records and assessments of tissue sections and classified into the following categories: systemic bleeding tendency, CNS involvement of leukemia, aspergillosis, and hemorrhagic infarction. ICH caused by CNS involvement was defined as the presence of dense leukemia cell infiltration in cerebral vessels at hemorrhagic sites with or without systemic bleeding tendency.

### 2.3 Immunohistochemical examination of leukemia cells and cerebral vessels

We focused on expression of hemostatic factors in leukemia cells. The primary antibodies used are described in Supplementary Table 1. Representative paraffin sections containing leukemia cells from each case were stained for VEGF (rabbit polyclonal, A-20; Santa Cruz Biotechnology), cathepsin G (rabbit, monoclonal, clone E3N30; Cell Signaling Technology), tissue-type plasminogen activator (tPA; rabbit polyclonal; GeneTex), urokinase-type plasminogen activator (uPA; rabbit polyclonal; Atlas Antibodies), urokinase-type plasminogen activator receptor (uPAR; mouse, monoclonal, clone E-3; Santa Cruz Biotechnology), and tissue factor (TF; mouse monoclonal, clone H-9; Santa Cruz Biotechnology). In cases without leukemia cells in the brain, representative paraffin blocks from other organs containing leukemia cells were selected. We defined a sample as immunopositive when >10% of leukemia cells were stained for VEGF, cathepsin G, tPA, uPA, uPAR, and TF, as previously reported [23]. Representative acute myeloid leukemia (AML) and acute lymphoblastic leukemia (ALL) cases were also stained by myeloperoxidase (MPO; rabbit, polyclonal; Dako/Agilent) and CD20 (mouse monoclonal, clone L26; Nichirei Biosciences), respectively. We then performed Sirius Red staining and immunohistochemistry to examine vascular integrity and permeability. Brain sections from the leukemia and control groups were stained for α-smooth muscle actin (SMA; mouse monoclonal antibody, clone 1A4; Dako/Agilent), fibrin (mouse monoclonal antibody, clone 59D8; EMD Millipore), and von Willebrand factor (VWF; mouse monoclonal, clone 36B11; Leica Biosystems). Sirius Red staining and immunohistochemistry for SMA were performed to assess vascular collagen fibers and smooth muscle cells, respectively. Collagen fibers were identified as regions exhibiting an orange-to-green color shift under a polarized lens. Vascular injury was defined as the disruption or fragmentation of SMA-positive or Sirius Red–positive layers. Abnormal vascular permeability was defined by the presence of fibrin or VWF deposition within the media and adventitia of the vascular wall or in the perivascular region. Sections were stained with EnVision, a dextran polymer conjugated with antimouse or antirabbit immunoglobulin and horseradish peroxidase (DAKO/Agilent). Horseradish peroxidase activity was visualized using 3,3'-diaminobenzidine solution containing hydrogen peroxide, and sections were counterstained with Mayer hematoxylin. Control sections were stained for nonimmune mouse or rabbit immunoglobulin. All histologic assessments were performed in a blinded manner.

We performed double immunofluorescence to examine cathepsin G and VEGF expression in leukemia cells in representative brain specimens. Brain sections were stained for MPO light chain (mouse monoclonal, clone A-5; Santa Cruz Biotechnology) and cathepsin G (clone E3N30) or VEGF (A-20). CF568 conjugated-donkey antimouse IgG (Biotium) and CF488 conjugated-donkey anti-rabbit IgG (Biotium) were used as secondary antibodies. Brain sections were mounted using 4,6'-diamidino-2-phenylindole–containing reagents. Fluorescent images were captured using an all-in-1 fluorescence microscope (BZ-X810; Keyence). Image acquisition and channel merging were performed using the microscope’s dedicated software (BZ-X Series Application).

### 2.4 Statistical analysis

Categorical data were analyzed using Fisher exact test. For contingency tables containing 0 values, Fisher exact test was used solely to calculate *P* values, and continuity correction was not applied because odds ratios (ORs) or CIs were not computed. Univariate analyses were performed to evaluate associations between each variable and the outcome of interest. Data are presented as medians and ranges. Since all data showed a nonnormal distribution, the Mann–Whitney U-test was used for the comparisons of 2 groups. Statistical analyses were performed using GraphPad Prism 8.43 (GraphPad Software) and JMP Student Edition 19.0.1 (JMP Statistical Discovery LLC). A *P* value of <.05 was considered to be significant.

## 3 Results

### 3.1 Clinicopathologic characteristics of leukemia cases

Table 1 summarizes the clinicopathologic characteristics of leukemia cases in autopsy records (*n* = 37). Median age was 58 years, with a male-to-female ratio of 3:1. A history of chemotherapy for leukemia was noted in 92% of cases. Leukemia subtypes included AML (*n* = 25, 68%), ALL (*n* = 7, 19%), and chronic myeloid leukemia in the blast phase (CML-BP; *n* = 5, 14%). CNS involvement of leukemia cells was clinically diagnosed in 8% (3/37) of cases. Specifically, each of the 3 cases was diagnosed based on different clinical findings as follows: (1) seizures, electroencephalography, and cerebrospinal fluid examination; (2) seizure episodes alone; and (3) cerebrospinal fluid examination and magnetic resonance imaging. Hyperleukocytosis at death (WBC count > 100,000/μL) was observed in 14% (5/37) of cases.

**Table 1:** Clinicopathologic findings of autopsy cases of leukemia (*n* = 37).

Clinical background	Value
Age (y)	58 (13-82)
Male	28 (75.7)
Chemotherapy	34 (91.9)
Leukemia type	
AML	25 (67.6)
M2	7 (18.9)
M3/APL	2 (5.4)
M5	5 (13.5)
Unclassified/unknown	11 (29.7)
ALL	7 (18.9)
CML, blast phase	5 (13.5)
Clinical diagnosis of CNS involvement	3 (8.1)
Hyperleukocytosis at death^a^	5 (13.5)
Laboratory findings^b^	
WBC (×10^3^/μL)	3.6 (0.1-302)
Hemoglobin (g/dL)	7.8 (5.1-12.7)
Platelets (×10^3^/μL)	16 (1-99)
Na (mmol/L)	137 (124-202)
K (mmol/L)	4.1 (2.7-44.0)
BUN (mg/dL)	25.4 (6.4-153.4)
Creatinine (mg/dL)	1.2 (0.2-8.5)
Total bilirubin (mg/dL)	1.5 (0.3-27.4)
AST (U/L)	37 (6-20,160)
ALT (U/L)	41 (3-3,030)
LDH (U/L)	809 (151-43,410)
PT (s), *n* = 27	14.3 (11.8-48.1)
APTT (s), *n* = 27	36.4 (24.2-153.9)
Fibrinogen (mg/dL), *n* = 28	265 (20-933)
FDP (μg/mL), *n* = 29	13.7 (5.0-411.1)
D-dimer (μg/mL), *n* = 8	32.6 (3.3-108.9)
Autopsy findings	
Nonremission state of leukemia	33 (89.2)
CNS involvement of leukemia cells	20 (54.1)
Intracranial hemorrhage	25 *(*67.6)
Cerebral infarction	3 (8.1)
Severe infection^c^	24 (64.9)
Fungal infection	10 (27.0)
Bacterial infection	5 (13.5)
Infection, not specified	9 (24.3)
Systemic hemorrhage	20 *(*54.1)
Multiple microthrombi^d^	5 (13.5)
DVT	0 (0.0)
PE^e^	4 (10.8)
NBTE	2 (5.4)
Fatal brain herniation	10 (27.0)

Values are *n* (%) or median (range).

ALL, acute lymphoblastic leukemia; AML, acute myeloid leukemia; ALT, alanine transaminase; APL, acute promyelocytic leukemia; APTT, activated partial thromboplastin time; AST, aspartate transaminase; BUN, blood urea nitrogen; CML, chronic myeloid leukemia; CNS, central nervous system; DVT, deep vein thrombosis; FDP, fibrin degradation product; LDH, lactate dehydrogenase; NBTE, nonbacterial thrombotic endocarditis; PE, pulmonary embolism; PT, prothrombin time; WBC, white blood cell.

a Hyperleukocytosis was defined as a WBC count of >100,000/μL.

b The number of available laboratory data varied among coagulation parameters because of missing records in older cases.

c Severe infection was defined as an infectious disease diagnosed clinically and pathologically, which was related to the cause of death.

d Multiple microthrombi was defined as the presence of microthrombi in >3 organs.

e All PE cases were caused by fungal emboli.

Laboratory data for the leukemia group are summarized in Table 1. The median interval between blood sampling and death was 1 day (range, 0-12 days). Patients showed severe thrombocytopenia (median, 16 × 10^3^/μL) and anemia (median, hemoglobin 7.8 g/dL), with widely variable leukocyte counts (median, 3.6 × 10^3^/μL; range, 0.1-302 × 10^3^/μL). Renal parameters were elevated (BUN, 25.4 mg/dL; creatinine, 1.2 mg/dL), while aspartate transaminase, alanine transaminase, and lactate dehydrogenase varied markedly. Coagulation data (available in subsets) indicated prolonged PT and activated partial thromboplastin time (APTT), reduced fibrinogen, and increased fibrin degradation products (FDPs) and D-dimer.

Autopsy findings are summarized in Table 1. A postmortem examination revealed that 89% of cases (*n* = 33) were in a nonremission state. Pathologic CNS involvement was noted in 54% (20/37). ICH and cerebral infarction were identified in 68% (25/37) and 8% (3/37) of cases, respectively. Severe infection was present in 65% (24/37) of patients, including fungal infections in 27% (10/37), bacterial infections in 14% (5/37), and infections, not specified in 24% (9/37). Detailed microbiological species are shown in Supplementary Table 2. Systemic hemorrhage and multiple microthrombi were detected in 54% (20/37) and 14% (5/37) of cases, respectively. No cases of deep vein thrombosis were identified. Pulmonary embolism occurred in 4 cases (11%), all resulting from fungal emboli. Nonbacterial thrombotic endocarditis (NBTE) was observed in 5% (2/37), with 1 case complicated by fatal hemorrhagic infarction. Fatal brain herniation due to ICH was identified in 27% (10/37) of cases. Among the 2 acute promyelocytic leukemia (APL) cases in the cohort, 1 case received treatment with the DCMP regimen (daunorubicin, cytarabine, 6-mercaptopurine, and prednisolone), prior to the introduction of all-*trans* retinoic acid (ATRA), resulting in death from systemic aspergillosis and ICH. The other received ATRA and died of severe pneumonia without ICH. No patients had an apparent history of head trauma preceding ICH, based on the clinical records.

### 3.2 Summary of control cohort and comparison with the leukemia group

Demographic and clinicopathologic characteristics of the control group are summarized in Supplementary Table 3 and compared with those of the leukemia group in Supplementary Table 4. The median age of the control group was 64.0 years (range, 18-76 years), and the male-to-female ratio was 3:1. No cases had a history of hematological malignancies or CNS diseases. Solid cancer–bearing status was observed in 45% of cases, and all cases had a history of chemotherapy. Systemic hemorrhage, pulmonary embolism, and NBTE were observed in 35%, 20%, and 0% of cases, respectively, with no significant differences compared with the leukemia group. Severe infection and a history of chemotherapy were more frequent in the leukemia group (*P* = .050 and *P* = .0002, respectively). Detailed microbiological species are shown in Supplementary Table 2. DVT was more common in the control group (*P* = .039). The interval between blood sampling and death was 2 days (range, 0-12 days). Regarding laboratory findings, leukocyte counts did not differ significantly between the 2 groups (median, 9.3 × 10^3^/μL in the control group vs 3.6 × 10^3^/μL in the leukemia group; *P* = .22). Hemoglobin and platelet levels were significantly lower in the leukemia group (hemoglobin: 7.8 vs 9.6 g/dL; *P* = .0019; platelets: 16 vs 155.5 × 10^3^/μL; *P* < .0001). Potassium levels were also lower in the leukemia group (4.1 vs 4.8 mmol/L; *P* = .0073), whereas sodium, BUN, creatinine, bilirubin, aspartate transaminase, and alanine transaminase showed no significant differences between the 2 groups. Lactate dehydrogenase levels were significantly higher in the leukemia cohort (809 vs 439.5 U/L; *P* = .024), consistent with increased tumor burden. Coagulation parameters, including PT, APTT, fibrinogen, FDP, and D-dimer, did not differ significantly between groups, although the leukemia group showed a trend toward elevated D-dimer levels (*P* = .057). Full details are provided in Supplementary Tables 3 and 4.

### 3.3 Pathologic findings in leukemia complicated by ICH

Supplementary Table 5 shows the clinicopathologic findings of leukemia cases complicated by ICH (*n* = 25). Among leukemia types, ICH was frequently observed in AML (*n* = 16, 64%). A nonremission state was noted in 92% (23/25) of cases, with CNS involvement of leukemia in 44% (11/25). The locations of ICH included the brain parenchyma (76%, 19/25), the subarachnoid space extending to the Virchow–Robin space (64%, 16/25), the intraventricular space (28%, 7/25), the subdural region (12%, 3/25), and multiple sites (60%, 15/25). Fatal brain herniation occurred in 40% (10/25) of cases. The causes of ICH were as follows: systemic bleeding tendency (52%, 13/25), CNS involvement of leukemia (32%, 8/25), aspergillosis (8%, 2/25), emboli from NBTE (4%, 1/25), and co-occurrence of mucormycosis and leukemia involvement (4%, 1/25).

### 3.4 Pathologic findings in leukemia with CNS involvement

Table 2 shows the characteristics of leukemia cases with pathologic CNS involvement (*n* = 20). Only 5% (1/20) of cases were clinically diagnosed with CNS involvement. Among the 3 cases clinically diagnosed with CNS involvement, 2 did not show pathologic CNS involvement at autopsy. Hyperleukocytosis at death was observed in 20% (4/20) of cases. The frequencies of CNS involvement confirmed at autopsy were 48% (12/25) in AML, 71% (5/7) in ALL, and 60% (3/5) in CML-BP. Leukemia cells were detected in the subarachnoid space extending to the Virchow–Robin space (95%, 19/20) (Figure 1B–D), the intravascular space (50%, 10/20) (Figure 1E), and the brain parenchyma (35%, 7/20) (Figure 1F). A single case (5%, 1/20) showed leukemia cells only within the intravascular space, accompanied by leukostasis. ICH was noted in 50% (10/20) of cases with CNS involvement and was accompanied by leukemia cell infiltration at hemorrhagic sites in 40% (8/20) of cases (Figure 1E–G). Leukostasis and leukemic nodules were each observed in 20% (4/20) of cases (Figure 1H–K). Three cases exhibited both leukostasis and leukemic nodules, while 1 case showed leukostasis alone and another showed only leukemic nodules. All leukemic nodules were located within hemorrhagic areas (Figure 1J, K). Leukostasis in 2 cases and leukemic nodules in 1 case were accompanied by hyperleukocytosis.

**Table 2:** Characteristics of leukemia cases with pathologic CNS involvement (*n* = 20).

Characteristic	Value
Clinical diagnosis of CNS involvement	1 (5.0)
Hyperleukocytosis at death^a^	4 (20.0)
Leukemia type	
AML	12 (60.0)
ALL	5 (25.0)
CML, blast phase	3 (15.0)
Sites of leukemia cells in CNS^b^	
Subarachnoid extending to the Virchow–Robin space	19 (95.0)
Intravascular space	10 (50.0)
Brain parenchyma	7 (35.0)
Intracranial hemorrhage	10 (50.0)
Accompanied by leukemia cell infiltration in hemorrhagic sites	8 (40.0)
Complicated with fatal brain herniation	4 (20.0)
Intracranial leukostasis	4 (20.0)
Complicated with hyperleukocytosis at death	2 (10.0)
Leukemic nodules	4 (20.0)
Complicated with hyperleukocytosis at death	1 (5.0)

Values are *n* (%).

ALL, acute lymphoblastic leukemia; AML, acute myeloid leukemia; CML, chronic myeloid leukemia; CNS, central nervous system.

a Hyperleukocytosis was defined as a white blood cell count of >100,000/μL.

b Cases involved multiple sites.

### 3.5 Expression of hemostasis-related factors in leukemia cells

**Figure 2:**
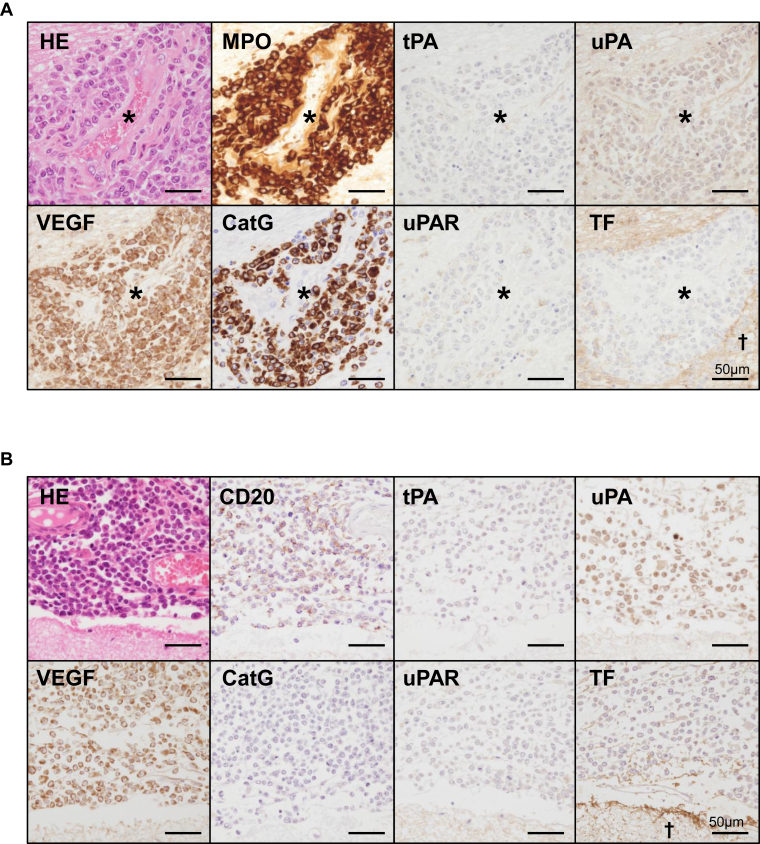

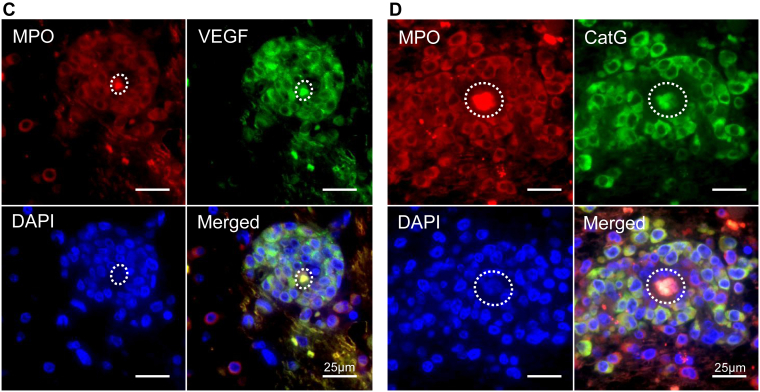
Expression of hemostatic factors in leukemia cells. (A) Leukemia cell infiltration in the Virchow–Robin space in a case of acute myeloid leukemia (AML). Asterisks indicate the vascular lumen. Myeloperoxidase (MPO)-positive leukemia cells express vascular endothelial growth factor (VEGF) and cathepsin G (CatG) but are negative for tissue-type plasminogen activator (tPA), urokinase-type plasminogen activator (uPA), urokinase-type plasminogen activator receptor (uPAR), and tissue factor (TF). (B) Leukemia cell infiltration in the subarachnoid space in a case of B cell acute lymphoblastic leukemia. CD20-positive leukemia cells express VEGF and uPA but are negative for CatG, tPA, uPA, uPAR, and TF. TF expression is observed in the surrounding glial tissue (dagger). (C, D) Double immunofluorescence of leukemia cells in the brain (same case as in Figure 2A). MPO-positive leukemia cells show the heterogeneous expression of VEGF (C) and CatG (D). Dashed lines indicate the vascular lumen. DAPI, 4′,6-diamidino-2-phenylindole.

Immunohistochemical expression of VEGF, cathepsin G, tPA, uPA, uPAR, and TF was examined in leukemia cells from 27 cases. Immunohistochemistry was performed on brain specimens with leukemia cells or on representative specimens from other affected organs (lymph node, spleen, bone marrow, kidney, or lung) if brain involvement was absent. Table 3 summarizes the frequency of immunohistochemical expression in leukemia cells according to CNS involvement and leukemia type. VEGF, cathepsin G, tPA, uPA, uPAR, and TF expression was observed in 85%, 33%, 4%, 78%, 11%, and 7% of cases, respectively (Table 3, Figure 2A, B). Among the leukemia types, VEGF and uPA were frequently expressed in all types. The expression of cathepsin G, a neutrophil serine protease, was observed in 41% of AML cases and in 50% of CML-BP cases, but not in ALL cases. In double immunofluorescence, MPO-positive myeloid leukemia cells heterogeneously expressed VEGF or cathepsin G (Figure 2C, D). TF was rarely expressed or absent in all leukemia types.

**Table 3:** Immunohistochemical expression of hemostatic factors in leukemia cells.

Leukemia type		VEGF	Cathepsin G	tPA	uPA	uPAR	Tissue factor
Total	*n* = 27	23 (85.2)	9 (33.3)	1 (3.7)	21 (77.8)	3 (11.1)	2 (7.4)
AML	*n* = 17	16 (94.1)	7 (41.2)	1 (5.9)	14 (82.4)	1 (5.9)	2 (11.8)
ALL	*n* = 6	4 (66.7)	0 (0.0)	0 (0.0)	4 (66.7)	0 (0.0)	0 (0.0)
CML, blast phase	*n* = 4	3 (75.0)	2 (50.0)	0 (0.0)	3 (75.0)	2 (50.0)	0 (0.0)
CLL/SLL	*n* = 27	23 (85.2)	9 (33.3)	1 (3.7)	21 (77.8)	3 (11.1)	2 (7.4)

Values are *n* (%).

ALL, acute lymphoblastic leukemia; AML, acute myeloid leukemia; CLL, chronic lymphocytic leukemia; CML, chronic myeloid leukemia; SLL, small lymphocytic lymphoma.

### 3.6 Vascular injury and abnormal permeability of cerebral vessels

**Figure 3:**
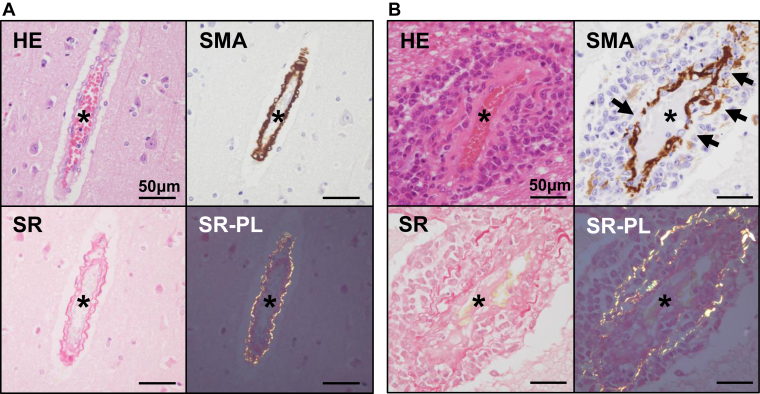

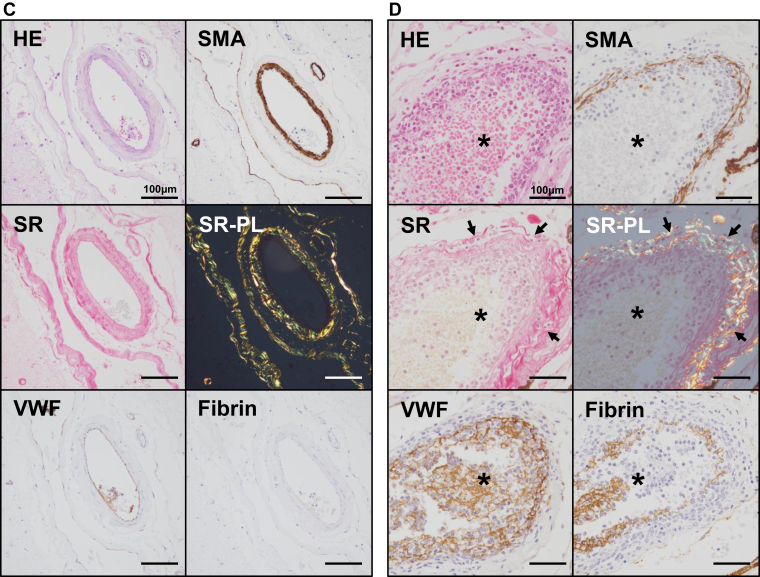
Direct vascular injury and abnormal permeability. (A) Histology of a cerebral vessel in the control brain. Smooth muscle layers and collagen fibers are highlighted by immunohistochemistry for smooth muscle actin (SMA) and Sirius Red (SR) staining, respectively. No disruption or fragmentation of SMA-positive smooth muscle cells or collagen fibers is observed. Additionally, no cellular infiltration is noted in the Virchow––Robin space. (B) Leukemia cell infiltration in the Virchow–Robin space in a case of acute myeloid leukemia (AML) (same case as in Figure 2A). In contrast to (A), SMA-immunopositive cells exhibit disruption and fragmentation, associated with leukemia cell involvement (arrows). Similarly, collagen fibers in the vessel wall, highlighted by SR, staining appear fragmented due to leukemia cell infiltration. Asterisks indicate the vascular lumen (A and B).

Since leukemia cells were characteristically observed along and within cerebral vessels, we examined vascular injury and abnormal permeability of cerebral vessels in the leukemia group (*n* = 37) and the control group (*n* = 20). Vascular injury was defined as the disruption or fragmentation of SMA-positive or Sirius Red–positive layers. Abnormal vascular permeability was defined by the presence of fibrin or VWF deposition within the media and adventitia of the vascular wall or in the perivascular region. The medial smooth muscle cell layer and adventitial collagen layer were preserved in the control group. Consequently, vascular injury was not observed in the control group (0/20) (Figure 3A). In contrast, vascular injury was detected in 35% (13/37) of leukemia cases. All cases with vascular injury were associated with the direct infiltration of leukemia cells into the vascular walls, either in the brain parenchyma or in the subarachnoid space (Figure 3B, D). No abnormal vascular permeability was noted in the control group (0/20) (Figure 3C). Abnormal vascular permeability was observed in 16% (6/37) of leukemia cases (Figure 3D).

### 3.7 Relationships among ICH, laboratory findings, clinicopathologic findings, and expression of hemostatic factors

We examined the relationships among ICH, CNS involvement, and the expression of hemostatic factors based on the data presented in Supplementary Table 6. Laboratory parameters were comparable between non-ICH and ICH groups, including WBC (median, 5.25 vs 3.6 × 10^3^/μL; *P* = .65), hemoglobin (7.35 vs 7.9 g/dL; *P* = .57), platelet count (15 vs 24 × 10^3^/μL; *P* = .36), PT (13.7 vs 14.55 seconds; *P* =.64), fibrinogen (361 vs 253.5 mg/dL; *P* = .13), FDP (8 vs 22.5 μg/mL; *P* = .26), and D-dimer (18.63 vs 73.01 μg/mL; *P* = .34). APTT tended to be prolonged in the ICH group (median, 33 vs 38 seconds), although this did not reach statistical significance (*P* = .065).

Clinicopathologic variables were generally comparable between non-ICH and ICH groups. The patients in the ICH group were younger (median age, 52 [13–76] vs 65.5 [38–82] years; *P* = .0056) than those in the non-ICH group (Supplementary Table 6). No significant differences were observed in sex, hyperleukocytosis at death, nonremission status, CNS involvement, severe infection, multiple microthrombi, vascular injury, or abnormal permeability between the 2 groups (Supplementary Table 6). All cases exhibiting pathologic leukostasis (*n* = 4) or leukemic nodules (*n* = 4) were found in the ICH group; however, their frequencies did not significantly differ between non-ICH and ICH groups (0% vs 33.3% for both; *P* = .12) (Supplementary Table 6). Hemostasis-related factors (TF, tPA, uPA, uPAR, cathepsin G, and VEGF) showed no significant differences between non-ICH and ICH groups; notably, uPA and VEGF were frequently expressed regardless of ICH status (Supplementary Table 6). Univariate logistic regression analysis for ICH revealed that, among laboratory findings, prolonged APTT (per 5 seconds; OR, 1.60; 95% CI, 1.02-3.12; *P* = .031) and lower fibrinogen levels (per 100 mg/dL; OR, 0.59; 95% CI, 0.28-1.00; *P* = .049) were associated with ICH (Supplementary Table 7).

### 3.8 Relationships among vascular injury, CNS involvement, and the expression of hemostatic factors

Among leukemia cases with CNS involvement (*n* = 20), cathepsin G expression was more frequent in cases with vascular injury than in those without vascular injury (54% vs 0%; *P* = .045) (Table 4). With respect to laboratory findings, cases with vascular injury showed a significantly prolonged APTT compared with those without vascular injury (median, 41 vs 27 seconds; *P* = .042) (Supplementary Table 8). In univariate logistic regression, APTT (per 5 seconds; OR, 2.5; 95% CI, 1.1-10.0; *P* = .015) was significantly associated with vascular injury (Supplementary Table 9).

**Table 4:** Relationships among vascular injury, CNS involvement, and the expression of hemostatic factors.

Leukemia-expressing factors in the brain^a^	Total (*n* = 20)	Nonvascular injury (*n* = 7)	Vascular injury (*n* = 13)	*P*
TF	0 (0)	0 (0)	0 (0)	1.0
tPA	1 (5.0)	1 (14.3)	0 (0)	.35
uPA	16 (80.0)	6 (85.7)	10 (76.9)	1.0
uPAR	1 (5.0)	0 (0)	1 (7.7)	1.0
Cathepsin G	7 (35.0)	0 (0)	7 (53.8)	.045
VEGF	18 (90.0)	6 (85.7)	12 (92.3)	1.0

Leukemia-expressing factors in the brain^a^	Total (*n* = 20)	Normal permeability (*n* = 16)	Abnormal permeability (*n* = 4)	*P*
TF	0 (0)	0 (0.0)	0 (0)	1.0
tPA	1 (5.0)	1 (6.3)	0 (0)	1.0
uPA	16 (80.0)	13 (81.3)	3 (75.0)	1.0
uPAR	1 (5.0)	0 (0)	1 (25.0)	.20
Cathepsin G	7 (35.0)	5 (31.3)	2 (50.0)	.59
VEGF	18 (90.0)	14 (87.5)	4 (100.0)	1.0

Leukemia-expressing factors in any organ^b^	Total (*n* = 27)	Normal permeability (*n* = 23)	Abnormal permeability (*n* = 4)	*P*
TF	2 (7.4)	2 (8.7)	0 (0.0)	1.0
tPA	1 (3.7)	1 (4.3)	0 (0.0)	1.0
uPA	21 (77.8)	18 (78.3)	3 (75.0)	1.0
uPAR	3 (11.1)	2 (8.7)	1 (25.0)	.39
Cathepsin G	9 (33.3)	7 (30.4)	2 (50.0)	.58
VEGF	23 (85.2)	19 (82.6)	4 (100.0)	1.0

Leukemia-expressing factors in any organ^b^	Total (*n* = 27)	Non-CNS involvement (*n* = 7)	CNS involvement (*n* = 20)	*P*
TF	2 (7.4)	2 (28.6)	0 (0.0)	.060
tPA	1 (3.7)	0 (0.0)	1 (5.0)	1.0
uPA	21 (77.8)	5 (71.4)	16 (80.0)	.63
uPAR	3 (11.1)	2 (28.6)	1 (5.0)	.16
Cathepsin G	9 (33.3)	2 (28.6)	7 (35.0)	1.0
VEGF	23 (85.2)	5 (71.4)	18 (90.0)	.27

Leukemia-expressing factors in any organ^b^	Total (*n* = 27)	Nonmeningeal invasion (*n* = 8)	Meningeal invasion (*n* = 19)	*P*
TF	2 (7.4)	2 (25.0)	0 (0.0)	.080
tPA	1 (3.7)	0 (0.0)	1 (5.3)	1.0
uPA	21 (77.8)	6 (75.0)	15 (78.9)	1.0
uPAR	3 (11.1)	2 (25.0)	1 (5.3)	.20
Cathepsin G	9 (33.3)	2 (25.0)	7 (36.8)	.68
VEGF	23 (85.2)	5 (62.5)	18 (94.7)	.065

Statistical analyses were performed using Fisher exact test. Values are *n* (%).

CNS, central nervous system; TF, tissue factor; tPA, tissue-type plasminogen activator; uPA, urokinase-type plasminogen activator; uPAR, urokinase-type plasminogen activator receptor; VEGF, vascular endothelial growth factor.

a Cases with viable leukemia cells in the brain (CNS involvement).

b Cases with viable leukemia cells in CNS or other organs.

Although the difference was not statistically significant, VEGF expression was more frequently observed in cases with meningeal invasion than in those without (95% vs 56%; *P* = .065) (Table 4). In univariate logistic regression, VEGF positivity was significantly associated with meningeal invasion (OR, 10.8; 95% CI, 1.12-247.26; *P* = .040) (Supplementary Table 10) Expression of other hemostatic factors did not significantly differ in cases with or without vascular injury, abnormal permeability, CNS involvement, or meningeal invasion (Table 4).

### 3.9 Relationship between the causes of ICH and pathologic findings

We also examined the relationship between causes of ICH and pathologic findings (Table 5). Vascular injury was significantly more frequent in ICH cases caused by leukemia cell infiltration than in those caused by bleeding tendency (75% vs 0%; *P* = .0005) (Table 5). However, no significant differences were observed in the frequency of abnormal permeability or expression of hemostatic factors in leukemia cells between ICH cases caused by bleeding tendency and those caused by CNS involvement (Table 5).

**Table 5:** Pathologic findings between causes of ICH.

Findings	Causes of ICH		
	Bleeding tendency (*n* = 13)	CNS involvement (*n* = 8)	*P*
Vascular injury (+)	0 (0)	6 (75)	.00050
Abnormal permeability (+)	1 (8)	2 (25)	.53

Leukemia-expressing factors in any organ^a^	Bleeding tendency (*n* = 6)	CNS involvement (*n* = 8)	*P*
TF	1 (16.7)	0 (0)	0.43
tPA	0 (0)	0 (0)	1.0
uPA	4 (66.7)	5 (62.5)	1.0
uPAR	1 (16.7)	1 (12.5)	1.0
Cathepsin G	1 (16.7)	4 (50.0)	.30
VEGF	3 (50.0)	8 (100)	.055

Statistical analyses were performed using Fisher exact test. Values are *n* (%).

CNS, central nervous system; ICH, intracranial hemorrhage; TF, tissue factor; tPA, tissue-type plasminogen activator; uPA, urokinase-type plasminogen activator; uPAR, urokinase-type plasminogen activator receptor; VEGF, vascular endothelial growth factor.

a Cases with viable leukemia cells in CNS or other organs.

## 4 Discussion

In this autopsy-based study, ICH occurred in 68% of leukemia cases, and CNS involvement was identified in 54%. Among the ICH cases, the underlying cause was attributed to systemic bleeding tendency in 52% and to direct infiltration of CNS by leukemia cells in 32% (Supplementary Table 5). Notably, 40% of patients with ICH developed fatal brain herniation. A histopathologic examination revealed that leukemia cell infiltration of meninges and vascular walls was associated with direct injury to cerebral vessel, and that vascular injury was frequently associated with leukemic infiltration-related ICH. Furthermore, leukemia cells frequently expressed VEGF, cathepsin G, and uPA. VEGF was highly expressed across all leukemia subtypes and was associated with meningeal invasion. In contrast, cathepsin G expression was primarily observed in myeloid leukemia and associated with vascular injury.

ICH is a major cause of mortality in leukemia [3]. Our results are consistent with previous findings showing the clinical severity of ICH in hematological malignancies [1,3,5]. Moreover, ICH in leukemia has been reported to involve various intracranial regions, including brain parenchyma, subarachnoid space, and subdural and epidural regions, as well as multifocal sites [1,24]. A clinical study demonstrated that multifocal cerebral hemorrhage in hematological malignancies was associated with a poor prognosis and that the mortality rate was high among patients with subarachnoid hemorrhage [1]. The present study also identified a high frequency of multiple-site ICH and subarachnoid hemorrhage, which is consistent with these findings [1,24]. Thrombocytopenia and coagulopathy are known risk factors for ICH [3]. The present study supports the notion, especially in prolonged APTT as a risk of ICH. In addition, prolonged APTT was also associated with cerebral vascular injury. In support of a potential mechanistic link, a retrospective cohort study reported a significantly higher incidence of ICH in patients with than in those without CNS involvement (46.1% vs 3%; *P* < .001) [25]. To further examine this relationship, we pathologically confirmed a high frequency of CNS infiltration by leukemia cells, characterized by expression of hemostasis-related factors. Moreover, the leukemia cell infiltration of vascular walls was accompanied by loss or degeneration of medial smooth muscle cells and adventitial collagen. Interestingly, leukemia cell infiltration was more frequently observed in the Virchow–Robin space surrounding blood vessels, and vascular injury defined by disruption of the media and adventitia was more frequently noted than abnormal vascular permeability reflecting endothelial cell injury. These results suggest that leukemia cell infiltration of vascular walls makes them vulnerable and that vascular injury predominantly occurs from the adventitial side of cerebral vessels in leukemia. These pathologic features were present regardless of the ICH status, suggesting that CNS involvement contributes to local vascular injury and increases the risk of ICH even in the absence of clinically evident ICH.

Autopsy-based studies on CNS lesions in leukemia were published in the 1960s and reported that blast crisis and hyperleukocytosis were associated with multiple cerebral hemorrhages and leukemia cell infiltration in CNS [12,13,15]. They also identified 2 characteristic patterns of leukemic involvement: leukostasis and leukemic nodules. In parallel with these early findings, the present study demonstrated that all cases with leukostasis or leukemic nodules were accompanied by ICH; however, the frequency of leukostasis and leukemic nodules did not significantly differ between ICH and non-ICH groups (0% vs 33.3% for both; *P* = .12). This may be due to the small number of cases examined in our study. Kawanami et al. [17] classified the causes of ICH as fungal embolism, leukemic infiltration, and coagulopathy based on the clinical course and autopsy findings [17]. Our analysis consistently identified systemic bleeding tendency, CNS involvement, fungal embolization, and NBTE as contributing factors. However, previous studies lacked a pathologic evaluation of hemorrhagic sites, vascular structures, and CNS involvement, particularly in comparisons with non-ICH or control leukemia cases. To the best of our knowledge, this is the first study to comprehensively evaluate these features in autopsy-confirmed leukemia and identify cerebral vessel injury by leukemia cells and abnormal permeability as novel pathologic findings in leukemia cases. The present results suggest that vascular injury, but not abnormal permeability, is associated with the pathogenesis of ICH related to CNS involvement of leukemia cells.

CNS involvement of leukemia has been reported in 30% to 80% of pediatric ALL [26], 30% to 40% of adult ALL [27], and 1.1% to 30% of AML cases [28,29]. It is also associated with a poor prognosis [[26], [27], [28]]. An autopsy study reported CNS involvement in 82% of ALL and 40% of AML cases [16]. In the present study, CNS involvement was observed in ∼60% of leukemia cases in a nonremission state at the time of death, and only 5% of these cases had a clinical diagnosis of CNS involvement. Therefore, CNS involvement may occur asymptomatically and potentially cause cerebral vascular injury.

Meningeal invasion is a major route of CNS involvement in leukemia. In a murine model, Yao et al. [30] demonstrated that leukemia cells infiltrated the subarachnoid space by migrating along blood vessels connecting the bone marrow of the skull and spine. Human autopsy studies also described leukemic localization within the meninges [13,15,16]. Moore [13] reported subarachnoid and perivascular infiltration in 30% and 19% of autopsy cases with acute leukemia, respectively. The present study revealed that 50% of leukemia cases exhibited meningeal invasion, specifically infiltration of the subarachnoid space extending to the Virchow–Robin space. However, direct vascular injury caused by meningeal leukemia cells is yet to be reported. In addition to these findings, the present study suggests that intravascular leukemia cells contribute to vascular damage and increased permeability from the luminal side. These observations raise the possibility that leukemia cells may infiltrate cerebral vessels from both outside (via perivascular spaces) and inside (via endothelium), potentially reflecting the unique anatomical structure of the brain. However, further studies are needed to confirm the route of leukemic cell infiltration into cerebral vessels.

VEGF expression has been reported in various leukemia cell lines [31]. In this study, we found that VEGF was frequently expressed by leukemia cells in the brain across various histologic types. VEGF plays a critical role in endothelial function and promotes vascular permeability, potentially leading to hemorrhage [31,32]. Cheng et al. [19] reported that VEGF-overexpressing glioblastoma cells induced ICH in a murine model. However, VEGF expression did not significantly differ in leukemia cells with or without abnormal permeability or vascular injury in this study. Tumor-derived VEGF also contributes to tumor growth and metastasis [31,32]. Moreover, high VEGF expression in leukemia cells was associated with meningeal invasion in an ALL murine model [33]. In that study, the transendothelial migration of leukemia cells was regulated by VEGF and contributed to CNS involvement [33]. The present study also revealed that VEGF expression in leukemia cells was more frequently observed in cases with than in those without meningeal invasion. Therefore, VEGF appears to affect the leukemia cell infiltration of meninges rather than vascular permeability and injury in humans.

Cathepsin G is a serine protease predominantly found in neutrophils and is involved in host defenses, regulation of inflammation, vascular homeostasis, and thrombosis [34]. It also contributes to tissue remodeling by degrading extracellular matrix proteins and activating matrix metalloproteinases [34]. Shamamian et al. [35] demonstrated that neutrophil-derived cathepsin G activated pro–matrix metalloproteinase 2 and induced microvascular damage *in vitro*. Furthermore, cathepsin G has been shown to affect the morphology of endothelial cells, inducing intercellular junctional disruption that increases vascular permeability [35]. Cathepsin G is expressed in leukemia cells, particularly in AML [20]. The present study demonstrated that cathepsin G was expressed in myeloid leukemia cells and vascular wall–infiltrating cells and was associated with vascular injury. uPA, a molecule involved in fibrinolysis and extracellular matrix degradation [36], was also frequently expressed by leukemia cells, as noted in the present study; however, it was not associated with vascular injury, abnormal permeability, CNS involvement, or meningeal invasion, and thus, its pathogenetic significance remains unclear. This is the first study based on human autopsy specimens to suggest that leukemia-expressed cathepsin G directly contributed to vascular injury within the brain. Expression of cathepsin G in leukemia cells may have potential value in predicting the risk of ICH especially in myeloid leukemia and could thus help inform patient management in the future.

This study has several limitations. Due to its retrospective design and reliance on autopsy specimens, laboratory data were not consistently available. We extracted values obtained closest to death; therefore, these results may not reflect the prehemorrhagic state and could have been influenced by terminal illness and/or therapeutic interventions. Along with the small sample size, these factors limited our ability to detect significant relationships and to correlate pathologic findings with hemostasis-related parameters. Furthermore, leukemia subtypes were classified using clinical and histologic data, without the full immunophenotypic or genetic information needed for classification under the 2022 World Health Organization criteria. Future studies integrating standardized laboratory and molecular data may provide further insights into the relationship between the CNS pathology and leukemia subtypes. Additionally, the small number of APL cases, a subtype with a high hemorrhagic risk, may limit the generalizability of our results to this population. The long study period (1977-2023) may also have introduced temporal bias due to changes in diagnostic standards and treatments. In fact, 1 APL case treated with chemotherapy, prior to the introduction of ATRA, developed ICH, whereas another APL case treated with ATRA did not develop ICH. These therapeutic and diagnostic changes over time may have influenced the observed frequencies of CNS involvement and ICH. Because of the small sample size and limited number of events, we reported univariable models only, which should be interpreted as supportive. The study was based on pathologic and immunohistochemical analysis and did not include mechanistic investigations.

In conclusion, this autopsy-based study revealed CNS involvement in a large percentage of leukemia cases that was frequently accompanied by ICH. Leukemia cell infiltration, via both meningeal and intravascular routes, was pathologically associated with cerebral vascular injury. Cerebral vascular injury may represent a mechanism of ICH associated with CNS involvement by leukemia cells. Importantly, we identified VEGF and cathepsin G as potential markers of meningeal invasion and cerebral vascular damage in leukemia, respectively. These results provide novel insights into mechanisms underlying CNS vascular pathology and hemorrhagic complications in leukemia.
