# Supplementary material for: Cathepsin G is associated with cerebral vascular injury in myeloid leukemia: a pathologic insight into intracranial hemorrhage
Source: Res Pract Thromb Haemost. 2026 Mar 26;10(3):103433. doi: 10.1016/j.rpth.2026.103433 (PMC13098595; doi:10.1016/j.rpth.2026.103433)
Supplement: Supplementary Figure and Tables [file mmc1.pdf]

SUPPLEMENTARY MATERIALS

Cathepsin G is associated with cerebral vascular injury in myeloid leukemia: A pathological insight into intracranial hemorrhage

Toshihiro Gi[1], Kaiyou Kai[1], Kotaro Shide[2], Eriko Nakamura[1], Nobuyuki Oguri[1], Murasaki Aman[1], Kazunari Maekawa[3], Sayaka Moriguchi-Goto[3], Michikazu Nakai[4], Kazuya Shimoda[2], Yohei Hisada[4], Atsushi Yamashita[1]*

[1]Department of Pathology, Faculty of Medicine, University of Miyazaki, Miyazaki, Japan
[2]Division of Hematology, Diabetes, and Endocrinology, Department of Internal Medicine, Faculty of Medicine, University of Miyazaki, Japan
[3]Department of Diagnostic Pathology, University of Miyazaki Hospital, Faculty of Medicine, University of Miyazaki, Miyazaki, Japan
[4]Department of Statistics and Data Management, Faculty of Medicine, University of Miyazaki, Miyazaki, Japan.
[5]UNC Blood Research Center, Division of Hematology, Department of Medicine, University of North Carolina at Chapel Hill, Chapel Hill, North Carolina, USA

***Corresponding author:**
Atsushi Yamashita, M.D., Ph.D.
Department of Pathology, Faculty of Medicine, University of Miyazaki, Miyazaki, Japan
5200 Kihara, Kiyotake, Miyazaki 889-1692, Japan
E-mail: atsushi_yamashita@med.miyazaki-u.ac.jp

Table S1. Primary antibodies for immunohistochemistry and immunofluorescence

Antibody	Antigen/Marker	Species	Clone	Dilution	HIER	Company	Catalog number
VEGF (A-20)	vascular endothelial growth factor	rabbit	polyclonal	1:500	MW	Santa Cruz Biotechnology	Sc-152
Cathepsin G	a neutrophil serine protease	rabbit	E3N30	1:500	MW	Cell Signaling Technology	P08311
tPA	tissue plasminogen activator	rabbit	polyclonal	1:100	MW	GeneTex	GTX103453
uPA	urokinase-type plasminogen activator	rabbit	polyclonal	1:50	MW	Atlas Antibodies	HPA008719
uPAR	urokinase-type plasminogen activator receptor	mouse	E-3	1:50	MW	Santa Cruz Biotechnology	Sc-376494
Tissue factor	tissue factor	mouse	H-9	1:200	MW	Santa Cruz Biotechnology	Sc-374441
SMA	α-smooth muscle actin, the median of vessels	mouse	1A4	1:200	(-)	Agilent/Dako	M0851
VWF	von Willebrand factor	mouse	36B11	1:100	MW	Leica Biosystems	NCL-L-vWF
Fibrin	fibrin	mouse	59D8	1:1000	MW	EMD Millipore Corp.	MABS2155-25-UG
Myeloperoxidase	myeloperoxidase	rabbit	polyclonal	1:1000	(-)	Agilent/Dako	A0398
MPO light chain	myeloperoxidase	mouse	A-5	1:200	MW	Santa Cruz Biotechnology	Sc-365436
CD20	a B-cell marker	mouse	L26	1:50	MW	Nichirei Biosciences	422741

HIER, heat-induced epitope retrieval methods; MW, microwave. MPO light chain is only used for immunofluorescence.

Table S2. Causative pathogens of severe infections, n (%)

**Control group (n=7)**			**Leukemia group (n=24)**		
Fungal infection	0	(0)	Fungal infection	10	(41.7)
Bacterial infection	5	(71.4)	*Aspergillus*	*6*	*(25.0)*
Acinetobacter baumannii and Enterobacter asburiae	*1*	*(14.3)*	*Aspergillus and Mucor*	*2*	*(8.3)*
Escherichia coli and Proteus vulgaris	*1*	*(14.3)*	*Candida*	*1*	*(4.2)*
Klebsiella pneumoniae	*1*	*(14.3)*	*Candida and Mucor*	*1*	*(4.2)*
Serratia marcescens	*1*	*(14.3)*	Bacterial infection	5	(20.8)
Methicillin-resistant Staphylococcus aureus (MRSA)	*1*	*(14.3)*	*Aeromonas hydrophila and Escherichia coli*	*1*	*(4.2)*
Infection, not specified	2	(28.6)	*Bacillus species*	*1*	*(4.2)*
			Bacteroides fragilis and Enterococcus avium	*1*	*(4.2)*
			Escherichia coli and Klebsiella pneumoniae	*1*	*(4.2)*
			Klebsiella pneumoniae	*1*	*(4.2)*
			Infection, not specified	9	(37.5)

Bacterial pathogens were identified based on antemortem blood culture results, whereas fungal pathogens were determined either by blood culture or by the morphological characteristics observed in autopsy specimens.

Table S3. Clinicopathological background of the control group (n=20)

**Clinical background**			**Laboratory findings, median (range)‡**		
Age, y, median (range)	64.0	(18-76)	WBC (×10³/μL)	9.3	(1.8-39.1)
Male, n (%)	15	(75.0)	Hb (g/dL)	9.6	(3.9-15.5)
Cancer-bearing status, n (%)	9	(45.0)	Plt (×10³/μL)	155.5	(11-587)
Primary organ of cancer, n (%)			Na (mmol/L)	135.5	(127-147)
Lung	*3*	*(15.0)*	K (mmol/L)	4.8	(3.2-11.0)
Gastrointestinal system	*3*	*(15.0)*	BUN (mg/dL)	36.2	(6.2-131.2)
Thyroid gland	*2*	*(10.0)*	Cre (mg/dL)	1.3	(0.2-5.6)
Skin	*1*	*(5.0)*	T. bil (mg/dL)	0.7	(0.2-28.8)
Chemotherapy, n (%)	9	(45.0)	AST (U/L)	61.5	(13-2,917)
Interstitial pneumonia, n (%)	3	(15.0)	ALT (U/L)	42	(4-894)
Systemic vasculitis, n (%)	1	(5.0)	LDH (U/L)	439.5	(149-5,497)
Severe infection, n (%)	7	(35.0)	PT (sec), n=18	13.25	(11.1-24.8)
Systemic hemorrhage, n (%)	7	(35.0)	APTT (sec), n=17	35.4	(23.5-59.3)
Multiple microthrombi, n (%)	0	(0)	Fibrinogen (mg/dL), n=13	368	(114-658)
DVT, n (%)	*3*	(15.0)	FDP (μg/mL), n=15	16.3	(2.8-124.2)
PE, n (%)	4	(20.0)	D-dimer (μg/mL), n=17	5.6	(0.9-57.7)
NBTE, n (%)	0	(0)			

Abbreviations: Cre, creatinine; DVT, deep vein thrombosis; NBTE, non-bacterial thrombotic endocarditis; PE, pulmonary embolism; T. bil, total bilirubin.

* Severe infection was defined as an infectious disease diagnosed clinically and pathologically, which was related to the cause of death.

† Multiple microthrombi was defined as the presence of microthrombi in more than three organs.

‡ The number of available laboratory data varied among coagulation parameters because of missing records in older cases.

Table S4. Comparative analysis of clinicopathological characteristics between control and leukemia groups

	Control group (n=20)		Leukemia group (n=37)		P value
**Clinicopathological findings**					
Age, y, median (range)	64	(18-76)	58	(13-82)	0.20
Male, n (%)	15	(75.0)	29	(78.4)	1.0
Chemotherapy, n (%)	9	(45.0)	34	(91.9)	**0.00020**
Severe infection, n (%)	7	(35.0)	24	(64.9)	0.050
Systemic hemorrhage, n (%)	7	(35.0)	20	(54.1)	0.28
Multiple microthrombi, n (%)	0	(0)	5	(13.5)	0.15
DVT, n (%)	3	(15.0)	0	(0.0)	**0.039**
PE, n (%)	4	(20.0)	4	(10.8)	0.43
NBTE, n (%)	0	(0)	2	(5.4)	0.54
**Laboratory findings, median (range)**					
WBC (×10³/µL)	9.3	(1.8-39.1)	3.6	(0.1–302)	0.22
Hb (g/dL)	9.6	(3.9-15.5)	7.8	(5.1–12.7)	**0.0019**
Plt (×10³/µL)	155.5	(11-587)	16	(1–99)	**<0.0001**
Na (mmol/L)	135.5	(127-147)	137	(124–202)	0.57
K (mmol/L)	4.8	(3.2-11.0)	4.1	(2.7–44.0)	**0.0073**
BUN (mg/dL)	36.2	(6.2-131.2)	25.4	(6.4–153.4)	0.51
Cre (mg/dL)	1.3	(0.2-5.6)	1.2	(0.2–8.5)	0.60
T. bil (mg/dL)	0.7	(0.2-28.8)	1.5	(0.3–27.4)	0.11
AST (U/L)	61.5	(13-2917)	37	(6–20,160)	0.18
ALT (U/L)	42	(4-894)	41	(3–3,030)	0.85
LDH (U/L)	439.5	(149-5,497)	809	(151–43410)	**0.024**
PT (sec)	13.25	(11.1-24.8), n=18	14.3	(11.8–48.1), n=27	0.13
APTT (sec)	35.4	(23.5-59.3), n=17	36.4	(24.2–153.9), n=27	0.62
Fibrinogen (mg/dL)	368	(114-658), n=13	265	(20–933), n=28	0.086
FDP (µg/mL)	16.3	(2.8-124.2), n=15	13.7	(5.0–411.1), n=29	0.62
D-dimer (µg/mL)	5.6	(0.9-57.7), n=17	32.6	(3.3–108.9), n=8	0.057

Abbreviations: Cre, creatinine; DVT, deep vein thrombosis; NBTE, non-bacterial thrombotic endocarditis; PE, pulmonary embolism; T. bil, total bilirubin.

Statistical analyses were performed using Fisher's exact test or the Mann–Whitney U test.

The number of available laboratory data varied among coagulation parameters because of missing records in older cases.

Table S5. Clinicopathological findings of leukemia cases with intracranial hemorrhage (n=25)

**Leukemia type, n (%)**		
AML	16	(64.0)
ALL	5	(20.0)
CML, blast phase	4	(16.0)
**Leukemia status, n (%)**		
Non-remission state of leukemia	23	(92.0)
CNS involvement of leukemia cells	*11*	*(44.0)*
**Sites of intracranial hemorrhage*, n (%)**		
Brain parenchyma	19	(76.0)
Subarachnoid extending to the Virchow-Robin space	16	(64.0)
Intraventricular space	7	(28.0)
Subdural	3	(12.0)
Multiple sites	15	(60.0)
Complicated with fetal brain herniation	10	(40.0)
**Causes of intracranial hemorrhage, n (%)**		
Bleeding tendency	13	(52.0)
CNS involvement of leukemia	8	(32.0)
Aspergillosis	2	(8.0)
Emboli from NBTE (hemorrhagic infarction)	1	(4.0)
Mucormycosis + CNS involvement of leukemia	1	(4.0)

*Cases involved multiple sites.

Table S6. Relationships among ICH, laboratory findings, clinicopathological findings and expression of hemostatic factors

**Laboratory findings, median (range)**	**Total (n=37)**		**non-ICH (n=12)**		**ICH (n=25)**		**P value**
WBC (×10³/µL)	3.6	(0.1–302)	5.25	(0.1-116.6)	3.6	(0.1-302.0)	0.65
Hb (g/dL)	7.8	(5.1–12.7)	7.35	(5.1-9.4)	7.9	(5.2-12.7)	0.57
Plt (×10³/µL)	16	(1–99)	15	(1-54)	24	(1-99)	0.36
PT (sec)	14.3	(11.8–48.1), n=27	13.7	(12.7-17), n=9	14.55	(11.8-48.1), n=18	0.64
APTT (sec)	36.4	(24.2–153.9), n=27	33	(24.8-41), n=9	38	(24.2-153.9), n=18	0.065
Fibrinogen (mg/dL)	265	(20–933), n=28	361	(126-933), n=10	253.5	(20-415), n=18	0.13
FDP (µg/mL)	13.7	(5.0–411.1), n=29	8	(5-73.7), n=11	22.5	(5-411.1), n=18	0.26
D-dimer (µg/mL)	32.6	(3.3–108.9), n=8	18.63	(6.07-33.96), n=4	73.01	(3.27-108.9), n=4	0.34

**Clinicopathological findings, n (%)**	**Total (n=37)**		**non-ICH (n=12)**		**ICH (n=25)**		**P value**
Age, y, median (range)	58	(13-82)	65.5	(38-82)	52	(13-76)	**0.0056**
Male, n (%)	28	(75.7)	8	(66.7)	20	(80.0)	0.43
Hyperleukocytosis at death, n (%)	5	(13.5)	1	(8.3)	4	(16.0)	1.0
Non-remission state, n (%)	33	(89.2)	10	(83.3)	23	(92.0)	0.58
CNS involvement, n (%)	20	(54.1)	8	(66.7)	12	(48.0)	0.32
Severe infection, n (%)	24	(64.9)	9	(75.0)	15	(60.0)	0.48
Multiple microthrombi, n (%)	5	(13.5)	1	(8.3)	4	(16.0)	1.0
Vascular injury, n (%)	13	(35.1)	5	(41.7)	8	(32.0)	0.72
Abnormal permeability, n (%)	6	(16.2)	1	(8.3)	5	(20.0)	0.64

(Continues)

(Continued)

Leukemia-expressing factors in any organ*, n (%)	Total (n=27)		Non-ICH (n=10)		ICH (n=17)		P value
TF	2	(7.4)	1	(10.0)	1	(5.9)	1.0
tPA	1	(3.7)	1	(10.0)	0	(0.0)	0.37
uPA	21	(77.8)	10	(100.0)	11	(64.7)	0.057
uPAR	3	(11.1)	1	(10.0)	2	(11.8)	1.0
Cathepsin G	9	(33.3)	3	(30.0)	6	(35.3)	1.0
VEGF	23	(85.2)	9	(90.0)	14	(82.4)	1.0
**Leukemia-expressing factors in the brain†, n (%)**	**Total (n=20)**		**Non-ICH (n=8)**		**ICH (n=12)**		**P value**
TF	0	(0.0)	0	(0.0)	0	(0.0)	1.0
tPA	1	(5.0)	1	(12.5)	0	(0.0)	0.40
uPA	16	(80.0)	8	(100.0)	8	(66.7)	0.12
uPAR	1	(5.0)	0	(0.0)	1	(8.3)	1.0
Cathepsin G	7	(35.0)	2	(25.0)	5	(41.7)	0.64
VEGF	18	(90.0)	7	(87.5)	11	(91.7)	1.0
**Leukemia-involvement in the brain†, n (%)**	**Total (n=20)**		**Non-ICH (n=8)**		**ICH (n=12)**		**P value**
Leukostasis	4	(20.0)	0	(0.0)	4	(33.3)	0.12
Leukemic nodule	4	(20.0)	0	(0.0)	4	(33.3)	0.12

Abbreviations: Cre, creatinine; DVT, deep vein thrombosis; NBTE, non-bacterial thrombotic endocarditis; PE, pulmonary embolism; T. bil, total bilirubin.

Statistical analyses were performed using the Mann–Whitney U test Fisher’s exact test.

*Cases with viable leukemia cells in CNS or other organs. †Cases with viable leukemia cells in the brain (CNS involvement).

Table S7. Univariate logistic regression analysis for ICH

Variable	Unit	n	OR	95% CI	P value
**Laboratory findings**					
WBC	per 1,000 /μL	37	1.01	1.00 - 1.03	0.23
Hb	per 1 g/dL	37	1.21	0.79 - 1.97	0.40
Plt	per 10 × 10^3 /μL	37	1.03	0.99 - 1.07	0.14
PT	per 1 sec	27	1.09	0.93 - 1.80	0.39
APTT	per 5 sec	27	1.60	1.02 - 3.12	**0.031**
Fibrinogen	per 100 mg/dL	28	0.59	0.28 - 1.00	**0.049**
FDP	per 5 µg/mL	29	1.10	1.00 - 1.31	0.070
D-dimer	per 1 µg/mL	8	1.05	1.00 - 1.16	0.072
**Leukemia-expressing factors**					
TF positive	positive vs negative	27	0.56	0.02 - 15.38	0.70
tPA positive	positive vs negative	27	NE	NE	NE
uPA positive	positive vs negative	27	NE	NE	NE
uPAR positive	positive vs negative	27	1.20	0.10 - 28.00	0.89
Cathepsin G positive	positive vs negative	27	1.27	0.24 - 7.62	0.78
VEGF positive	positive vs negative	27	0.52	0.02 - 4.80	0.58

NE, not estimable because the model failed to converge.

Table S8. Relationships among cerebral vascular injury and laboratory findings

Laboratory findings, median (range)	Non vascular injury (n=7)		Vascular injury (n=13)		P value
WBC (×10³/μL)	11.2	(0.4-140.5)	11.4	(0.1-302)	0.86
Hb (g/dL)	7.1	(5.1-10.3)	8.3	(7.2-12.7)	0.078
Plt (×10³/μL)	27	(9-77)	16	(1-99)	0.69
PT (sec)	12.9, n=5	(12.8-14.4)	14.9, n=9	(11.8-48.1)	0.14
APTT (sec)	27, n=5	(24.2-36.2)	41, n=9	(24.8-153.9)	**0.042**
Fibrinogen (mg/dL)	245, n=5	(204-488)	204, n=8	(20-509)	0.44
FDP (μg/mL)	17.5, n=4	(5-46)	15, n=11	(5-411)	0.32
D-dimer (μg/mL)	31.2, n=1	(31.2)	65.8, n=4	(6.1-108.9)	NE

NE, not estimable due to small sample size.

Statistical analyses were performed using the Mann–Whitney U test.

The number of available laboratory data varied among coagulation parameters because of missing records in older cases.

Table S9. Univariate logistic regression analysis for vascular injury

Variable	Unit	n	OR	95% CI	P value
**Laboratory findings**					
WBC	per 1,000 /µL	20	1.0	0.99 - 1.02	0.78
Hb	per 1 g/dL	20	2.0	0.95 - 6.24	0.069
Plt	per 10 × 10^3 /µL	20	0.998	0.96 - 1.04	0.90
PT	per 1 sec	14	2.06	0.99 - 7.73	0.056
APTT	per 5 sec	14	2.5	1.11 - 10.01	**0.015**
Fibrinogen	per 100 mg/dL	13	0.73	0.29 - 1.63	0.44
FDP	per 5 µg/mL	15	1.09	0.97 - 1.47	0.28
D-dimer	per 1 µg/mL	5	1.02	0.96 - 1.16	0.48
**Leukemia-expressing factors**					
TF positive	positive vs negative	20	NE	NE	NE
tPA positive	positive vs negative	20	NE	NE	NE
uPA positive	positive vs negative	20	0.56	0.02 - 5.57	0.63
uPAR positive	positive vs negative	20	NE	NE	NE
Cathepsin G positive	positive vs negative	20	NE	NE	NE
VEGF positive	positive vs negative	20	2.0	0.07 – 56.87	0.65

NE, not estimable because the model failed to converge.

Table S10. Univariate logistic regression analysis for meningeal invasion

Variable	Unit	n	OR	95% CI	P value
TF	positive vs negative	27	NE	NE	NE
tPA	positive vs negative	27	NE	NE	NE
uPA	positive vs negative	27	1.25	0.15 - 8.41	0.82
uPAR	positive vs negative	27	6.0	0.49 - 143.59	0.16
Cathepsin G	positive vs negative	27	1.75	0.30 - 14.24	0.55
VEGF	positive vs negative	27	10.8	1.12 - 247.26	**0.040**

NE, not estimable because the model failed to converge.

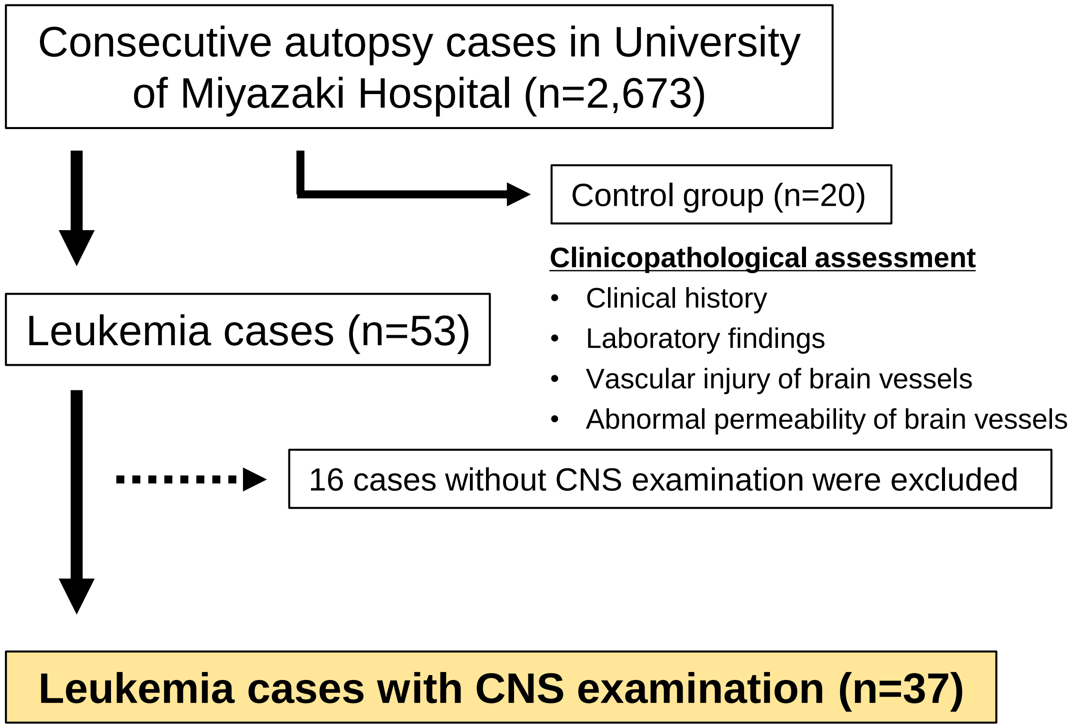

Analysis of autopsy-record summary (n=37)

- Clinical history
- Laboratory findings
- Leukemia classification
- Presence/localization of hemorrhage in CNS

Histopathological assessment

- Presence/localization of leukemia cell in CNS (n=37)
- Vascular injury of brain vessels (n=37)
  - Smooth muscle actin (IHC) and Sirius-red stain (for collagen fiber)
- Abnormal permeability of brain vessels (n=37)
  - Perivascular deposition of fibrin or von-Willebrand factor (IHC)
- Expression of hemostatic/ vascular factors and protease in leukemia cells (n=27)
  - VEGF, cathepsin G, tPA, uPA, uPAR, tissue factor (IHC)
- Double immunofluorescence (representative cases)

Figure S1. Summary of study design
